# Supplementary figures and images for: Social determinants of health among noncitizen deported US veterans: A participatory action study
Source: PLOS Glob Public Health. 2023 Aug 2;3(8):e0002190. doi: 10.1371/journal.pgph.0002190 (PMC10396001; doi:10.1371/journal.pgph.0002190)

**S1 Data.** “At the Pawn Shop”


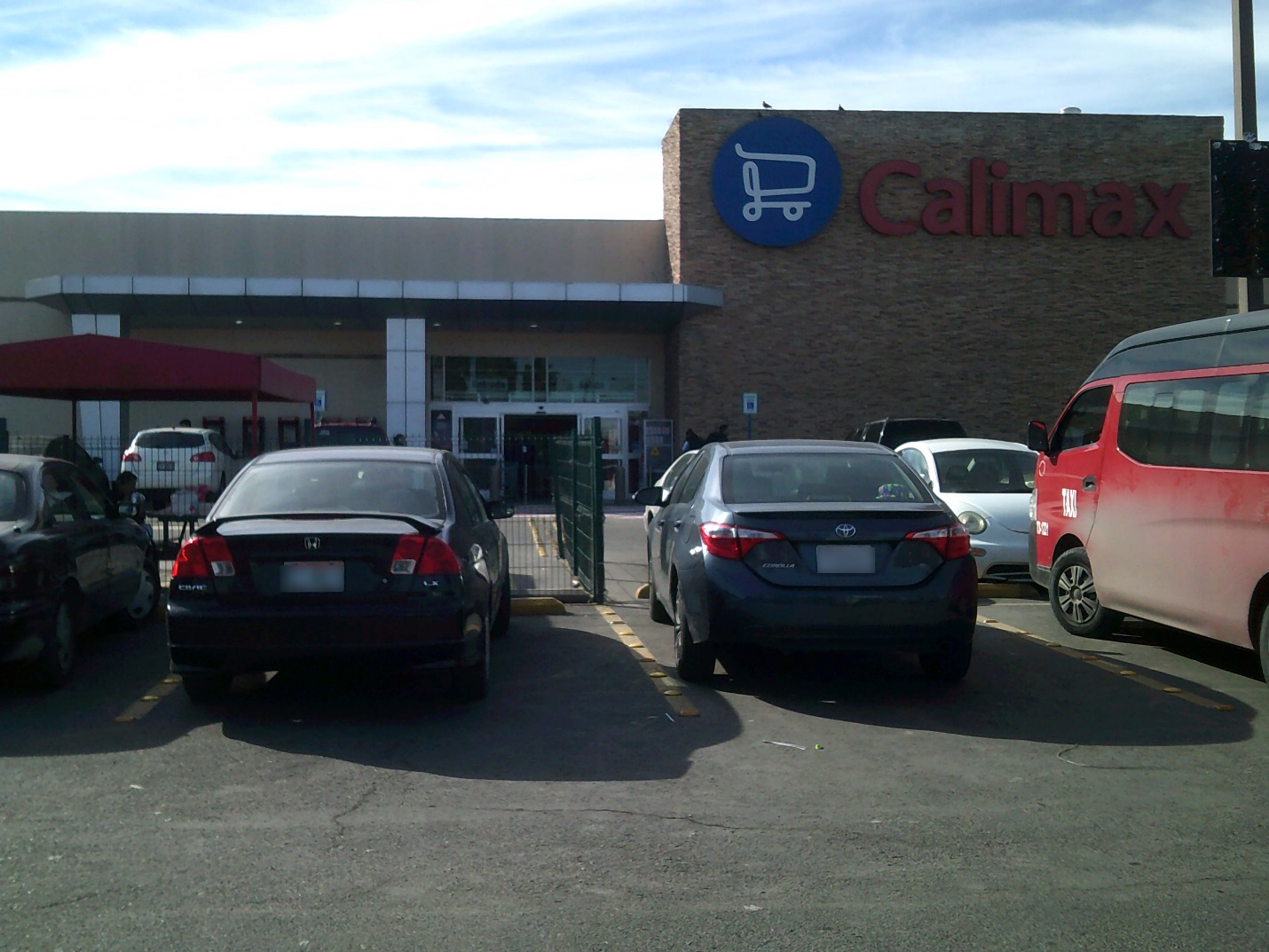

Supplement: S1 Data — (DOCX) [file pgph.0002190.s001.docx]

**S2 Data.** “Hello, my name is Johnny. How may I help you?”


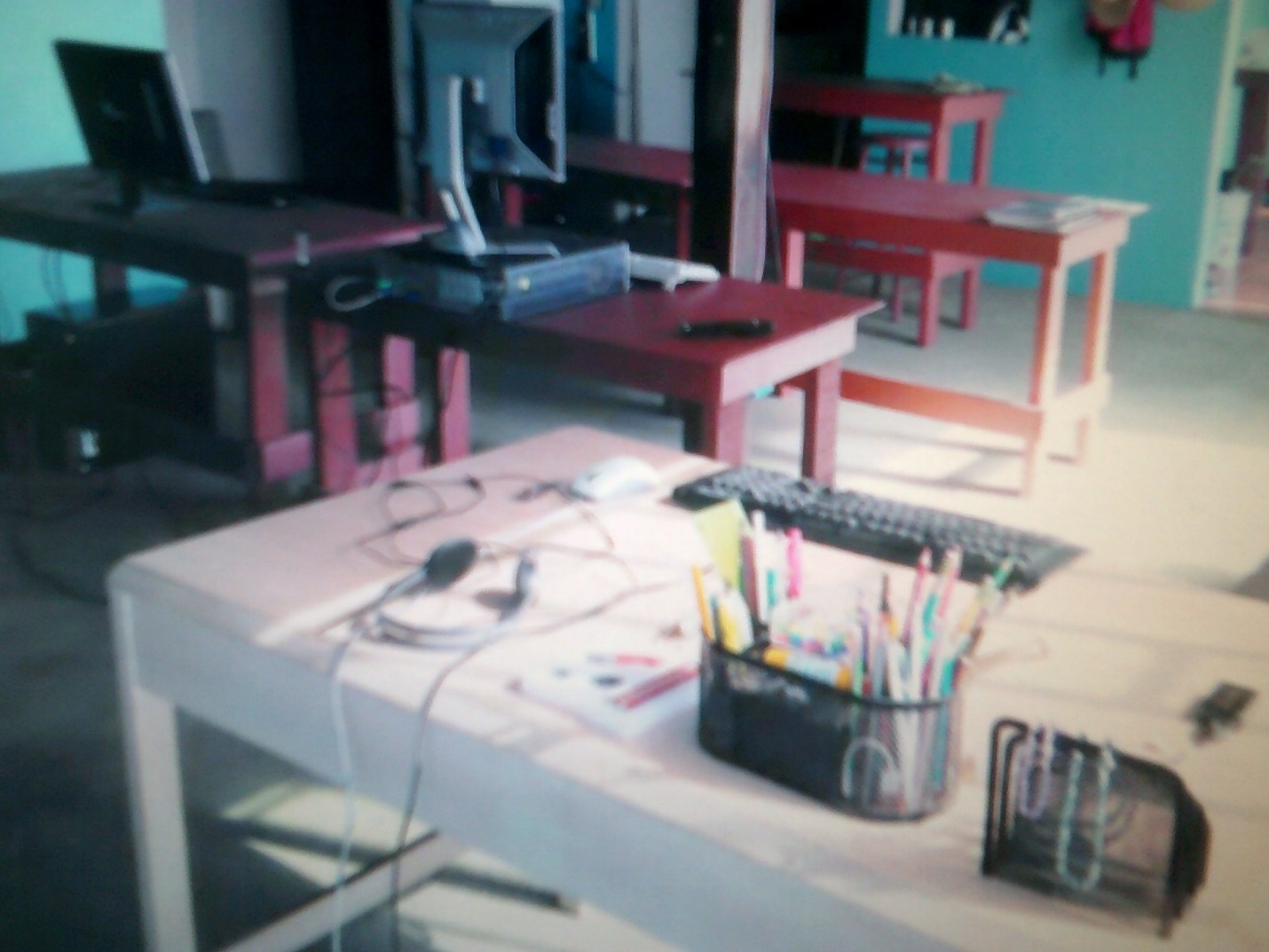

Supplement: S2 Data — (DOCX) [file pgph.0002190.s002.docx]

**S3 Data.** “On the Go in Mexico”

**
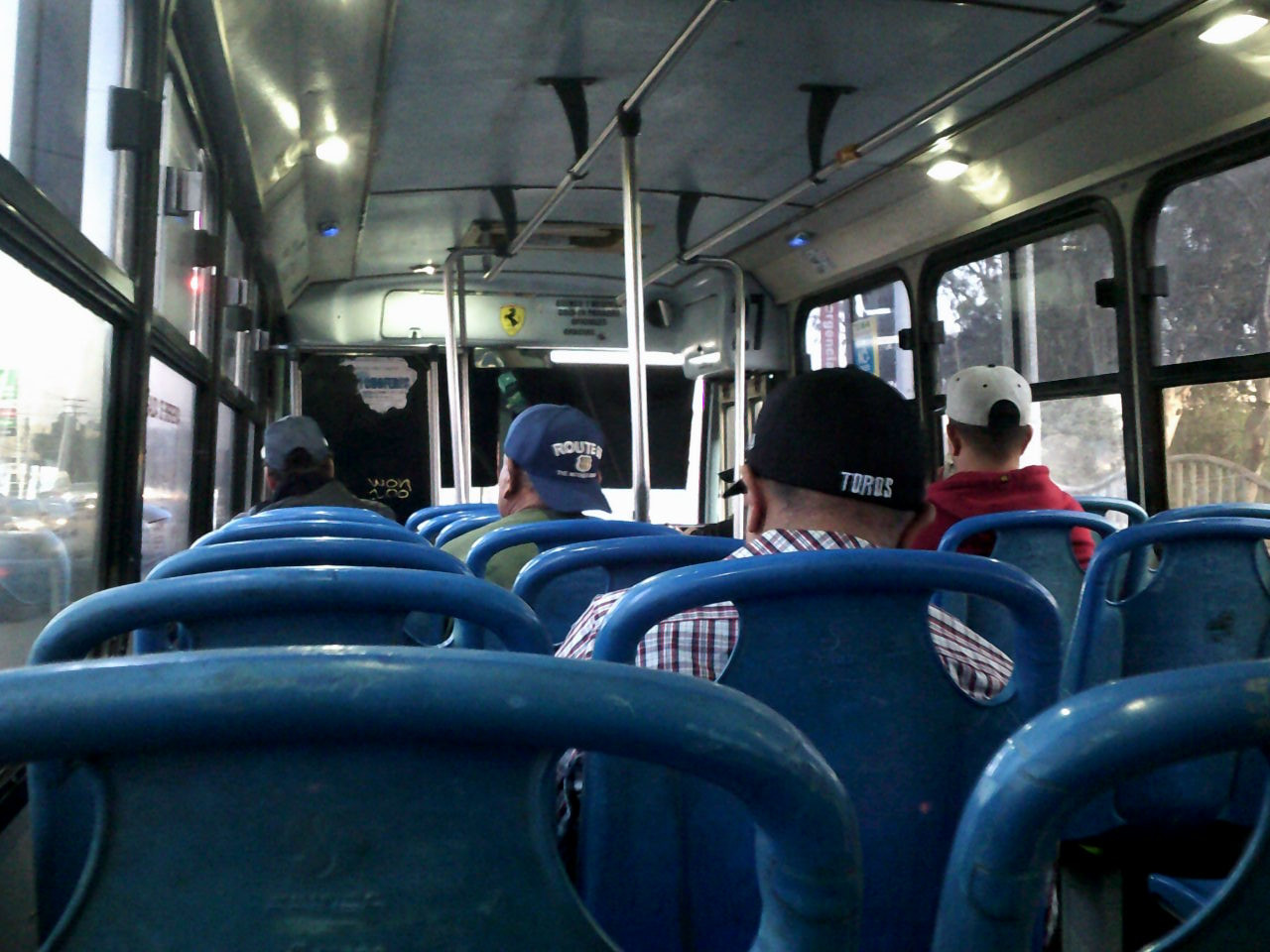
**

Supplement: S3 Data — (DOCX) [file pgph.0002190.s003.docx]

**S4 Data.** “My Life in Crumbles”

**
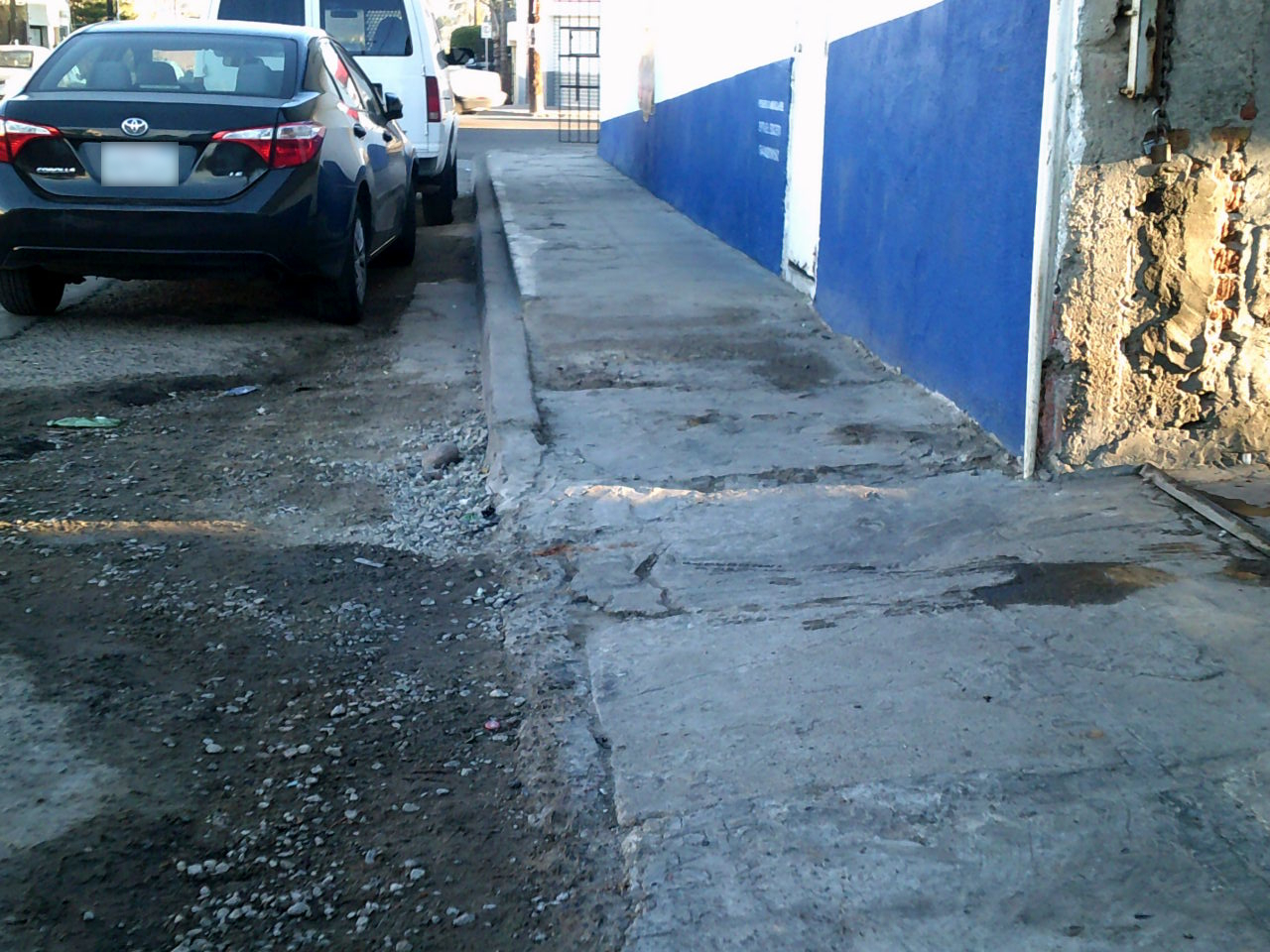
**

Supplement: S4 Data — (DOCX) [file pgph.0002190.s004.docx]

**S5 Data.** “What Hurts the Most”

**
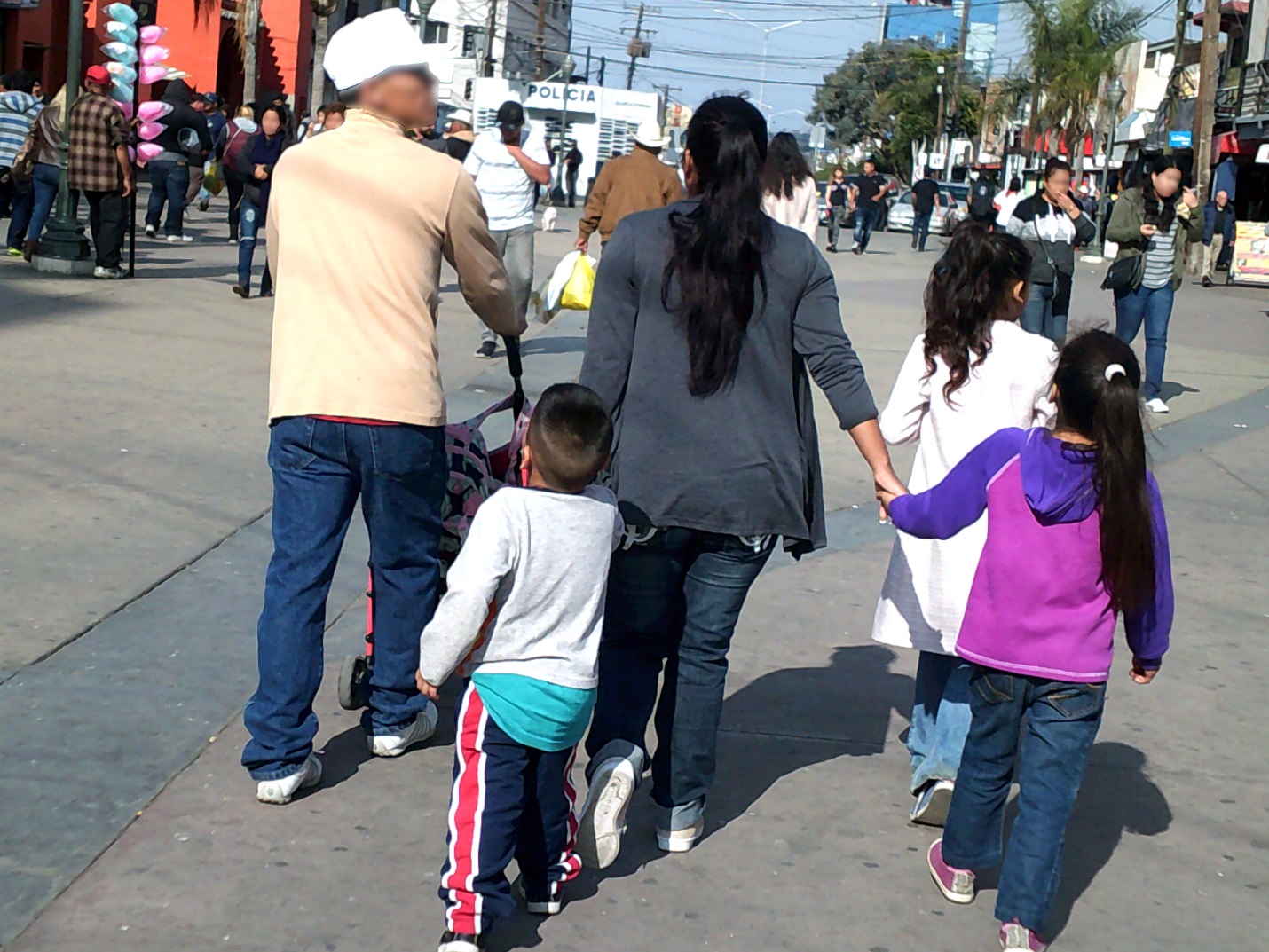
**

Supplement: S5 Data — (DOCX) [file pgph.0002190.s005.docx]

**S6 Data.** “Bleeding Out”


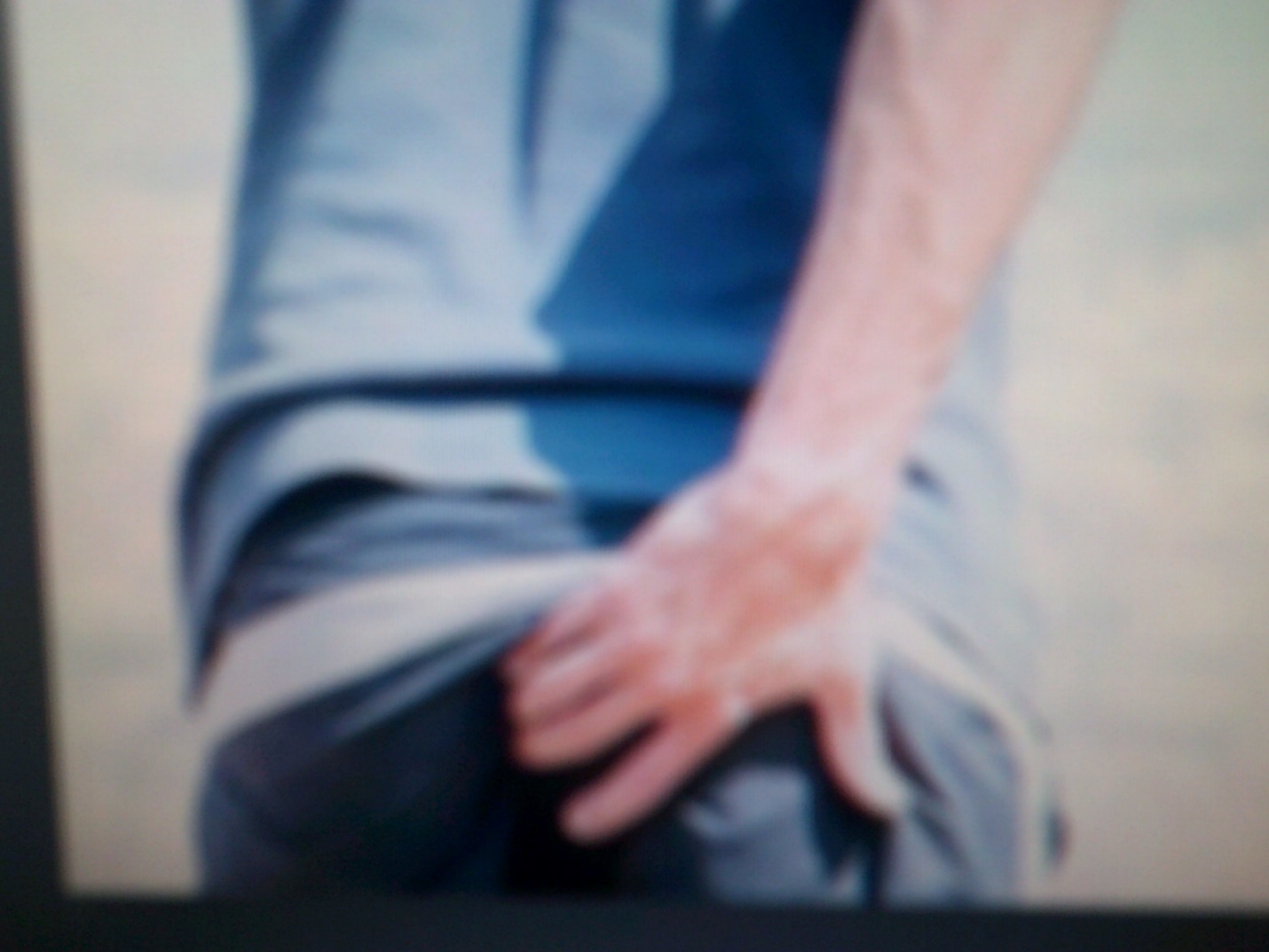

Supplement: S6 Data — (DOCX) [file pgph.0002190.s006.docx]

**S7 Data.** “Taking Away the Pain”


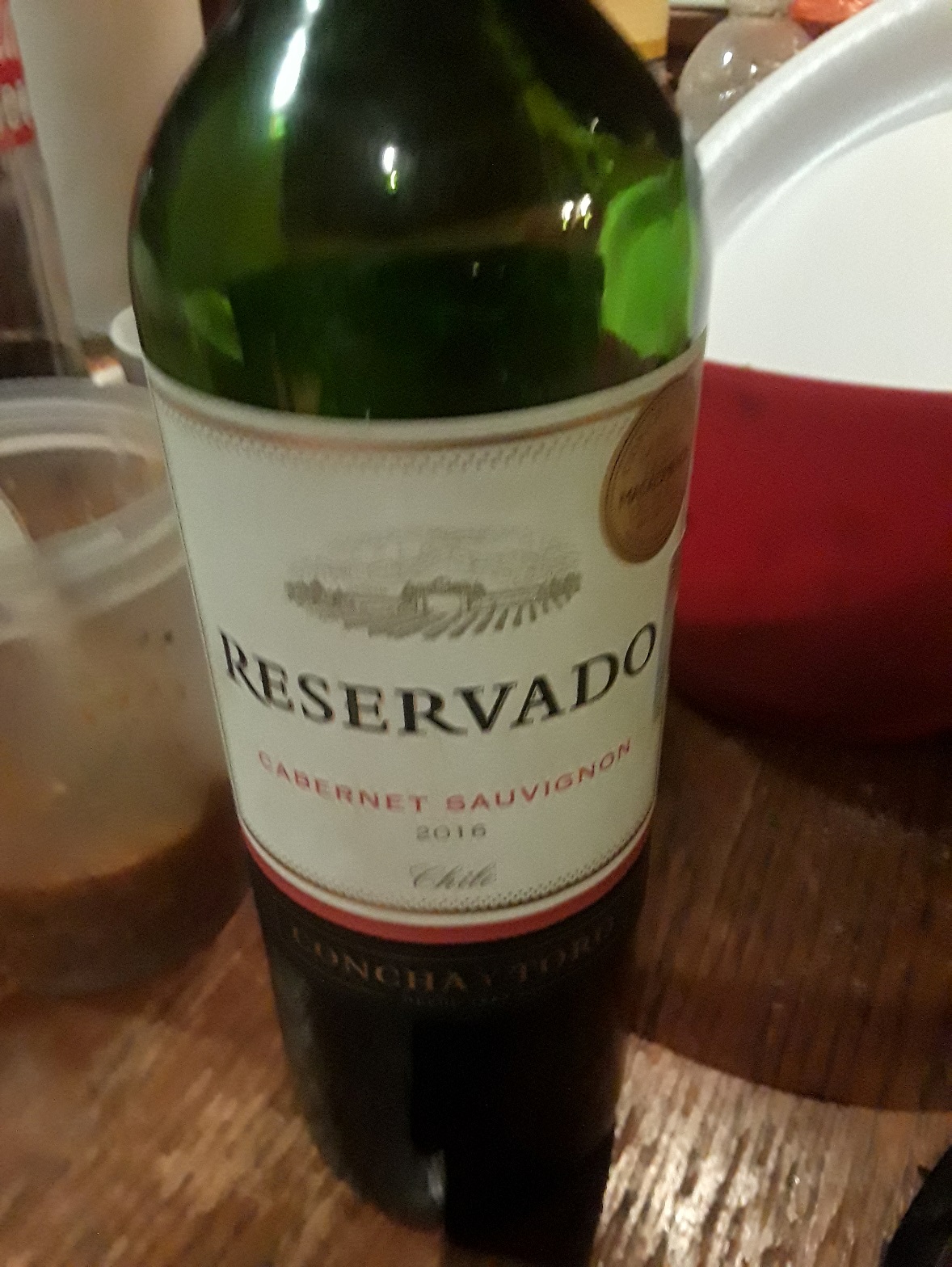

Supplement: S7 Data — (DOCX) [file pgph.0002190.s007.docx]
