# Supplementary material for: Social determinants of health among noncitizen deported US veterans: A participatory action study
Source: PLOS Glob Public Health. 2023 Aug 2;3(8):e0002190. doi: 10.1371/journal.pgph.0002190 (PMC10396001; doi:10.1371/journal.pgph.0002190)
Supplement: S1 Text — (PDF) [file pgph.0002190.s008.pdf]

IRB-SB approval is effective from date of this notice and good for the date indicated. Reviews are required to keep project active.

UNIVERSITY OF CALIFORNIA, RIVERSIDE
IRB Socio-Behavioral (IRB-SB)
Office of Research Integrity
August 13, 2018

APPROVAL NOTICE

INVESTIGATOR: Cheney, Ann; Lee, Cassidy

Faculty Advisor: Cheney, Ann

ACADEMIC UNIT: School of Public Policy

Administrator: n/a

PROJECT TITLE: "Using Qualitative Methods to Drive Health and Immigrant Policy Change"

IRB-SB. NUMBER: **HS - 18-128**

APPROVAL DATE: August 13, 2018

EXPIRATION DATE: August 12, 2020

FUNDING SOURCE: UC Mexus

SPECIAL CONDITIONS: None

THE UCR IRB-SB HAS REVIEWED THE PROPOSED USE OF HUMAN PARTICIPANTS IN THE REFERENCED APPLICATION AND APPROVED IT BASED ON THE FOLLOWING DETERMINATIONS:

1. Level of Review - 45 CFR 46.110 (#7) Expedited
2. Special Population - None
3. Risk - Minimal
4. The risks to participants are minimized by using procedures consistent with sound research design that do not unnecessarily expose participants to risk.
5. The risks are reasonable in relation to the anticipated benefits to individual participants and the importance of the knowledge that may reasonably be expected to result.
6. The selection of participants is reasonable and equitable.
7. The PI has had the appropriate human subjects research training.
8. Consent - Signed Consent Approved

Once the special conditions, if any, have been met, the protocol will be approved *through* August 12, 2020.

A "Continuing Review of the Approved Human Subjects Protocol" form will be sent to the PI three months before the expiration date, which will allow the PI to indicate whether to keep the application active or not. Please note that the expiration date is the last date that the application is approved.

<u>THE INVESTIGATOR SHALL PROMPLTY REPORT THE FOLLOWING TO THE IRB-SB:</u>
(1) Changes to the application (e.g., increase the number of participants, or changing the participant population, recruitment methods, procedures, documents) via an amendment, or
(2) Unanticipated problems involving risk to participants or others (please contact the IRB-SB for instructions).

DATE APPROVED August 13, 2018

[signature] pp

DR. DERICK FAY, CHAIR, IRB-SB
DESIGNATED UCR IRB-SB MEMBER
DR. MICHAEL PAZZANI (UCR IO), VICE CHANCELLOR, RESEARCH

May 29th, 2018

Cassidy T. Lee

University of California, Riverside

900 University Ave.

Riverside, CA 92521

Dear Mr. Lee,

The Deported Veteran Support House (DVSH) is pleased to learn about the opportunity to collaborate with the University of California, Riverside School of Public Policy and School of Medicine's Center for Healthy communities. We support the proposed research project titled "Veterans First, Deported Second: Community Review Board."

The DVSH is dedicated to supporting the health and well-being of deported U.S. veterans and especially in Mexico, and we share your concerns that U.S. veterans' health are being impacted by deportation. We are pleased to know that the aims of this study will elicit community feedback on this concern in our community and inform the development of future research.

Our organization will gladly work with you to determine the specifics of the Community Review Board that you plan to hold at the DVSH in July 2018. It is our understanding that we will assist you by verbally informing those we think may be interested in the attending and participating in the Community Review Board. We also agree to provide you with the potential participant's contact information should they give us permission.

The Deported Veteran Support House looks forward to participating and providing community members with this great opportunity. We are with high hopes that this Community Review Board will invite future research and trusted relationships with the deported veteran community.

Sincerely,

Hector Barajas, Founder

Deported Veteran Support House (DVSH)

Calle Calzada Tecnolojico near El Colejio Tecnolojico

Calle Juan Alvarez

1821 Fracc. Thomas Aquino, Tijuana BC Mexico
