# Supplementary material for: Social determinants of health among noncitizen deported US veterans: A participatory action study
Source: PLOS Glob Public Health. 2023 Aug 2;3(8):e0002190. doi: 10.1371/journal.pgph.0002190 (PMC10396001; doi:10.1371/journal.pgph.0002190)
Supplement: Attachment — Submitted filename: response to reviewer feedback.docx [file pgph.0002190.s009.docx]

Response to Reviewers

Manuscripts

PGPH-D-22-00720
Social Determinants of Health among Noncitizen Deported US Veterans: A Participatory Action Study
PLOS Global Public Health

Editor’s comments

1) The topic is of great interest and suitable for publication. However, there is a need of improving the manuscript. Discussion and conclusion should be re-elaborated based on reviewer comments.

Response: We have revised the discussion and conclusion to include the reviewers’ feedback, which has greatly improved this section of the paper and illustrated the public health impact.

2) The procedure of photovoice should be better explained, as well as the justification of the planned meetings during it. How do the authors intend to help participants to be heard by policymakers? Explain better how the analysis of the images and audio recording were.

Response: We have provided additional information about the photovoice method including information about the process and types of data collected, as well as a justification for the use of this method. We also include information on how the findings from this project informed a symposium on veteran health where policy makers and other key stakeholders participated and attended.

**Reviewer #1**

Thank you very much for requesting my review on the manuscript tilted " Social Determinants of Health among Noncitizen Deported US Veterans: A Participatory Action Study". This is a topic of major importance from both public health and health equity perspectives. The methodology was comprehensive in utilizing more than one qualitative tool to get information which gives more validity to the results. Additionally, engaging community in planning is proven to enhance the output with deeper insight due to gaining trust of the target population.

Response: Thank you for this positive feedback.

1) Quantitative data utilization. Although, it was declared as a limitation of the study, quantitative data analysis, literature shows that the minimum sample to be considered for quantitative analysis ranges between 20-40 minimum and without a probability sampling. Additionally, the statistical mean comparison between two populations with huge number difference does not provide stable nor robust results even if results would be weighted. Therefore, I would highly recommend to eliminate the quantitative analysis from the manuscript and adjust the results and discussion accordingly. This adjustment should not harm the concept and results since the key findings were extracted from the interviews and photo voice. This study would be highly valued as a qualitative study supported with interviews and photo voice tools.

Response: Thank you for this feedback. We agree that the qualitative data are strong and the quantitative analysis is not necessary for this paper. As such, we have eliminated the quantitative analysis from the manuscript and revised the results and discussion accordingly.

2) This study with all the effort done by the authors and collaborators, cannot provide a conclusive result that deportation "is a social determinant of health". However, it could suggest deportation as a possible determinant to be studied further in future research. Conclusive results demand different methodologies, sample size and analysis.

Response: We have revised this statement to clarify that our findings suggest deportation as a social determinant of health.

Reviewer #2: Background:

1) The first sentence seems at odds with the rest of the paragraph – perhaps adjust to “The US military may provide a fast track to citizenship….”

Response: We revised the first sentence to address this recommendation: “A common misnomer in the United States (US) is that military service provides a fast track to citizenship.”

2) I would like to see a better link between the paragraphs in the background. Jumping straight from citizenship to discharge to health consequences doesn’t flow well, I think the authors are endeavoring to link the implications of post-discharge behaviors and potential deportation, and flow on effects resulting from the deportation, it’s just a little clunky as is. This section would benefit from bridging sentences or paragraph to demonstrate the link between the concepts. Alternatively, an introductory paragraph that announces the concepts and their links before moving into the separate concepts.

Response: Thank you for this feedback. We have revised the background section by including transitions between paragraphs and ideas.

3) Methods: The “community review board” language is a little confusing in the same paragraph as the IRB – is there a different term that might be used to differentiate their purpose?

Response: We have clarified the discussion of the community review board. We also placed the discussion of IRB in a separate paragraph.

4) Pg 9: The manuscript directs to Table 1 for SF-36 scores and comparisons with the general population. Table 1 does not contain any of the SF-36 survey criteria, nor does it show population comparison. I would also question that the general US population is the best comparison group for a very small military cohort, many of whom experienced life changing events during service that would not occur in the general population, and all of whom were incarcerated.

Response: We have omitted the SF-36 scores and comparisons on this manuscript as per Reviewer 1’s recommendations.

5) Table 1 is also confusing in regard to the % column – % is not relevant to some items (eg mean age, age at first entry, year enlisted, year discharged etc) so it is unclear what the number in brackets is in these cases. I think it is referring to SD, but the table needs to be reformatted for clearer understanding.

Response: We reformatted Table 1 so that it’s clear we are referring to the percentages or the mean, SD.

6) Results/Discussion:
   a. The methods described do not relate to the results reported.

Response: We eliminated reference to the quantitative data collection, i.e., SF-36 so that the qualitative data, including narrative texts and photos are the primary data source in this manuscript. We also kept Table 1 for the purposes of presenting the socio-demographic characteristics of the qualitative sample (n=12).

b. Some of the language is opinion, subjective, and emotive, and there does not appear to be evidence to back the position taken.

Response: We revised any language that might be perceived as opinion, subjective or emotive.

c. I’m sure there was substantial qualitative data collected, so there may be a better way to report the information/data so that the results are more demonstrable and robust, rather than what appears to be author interpretation.

Response: We clarify that this is a qualitative study as well as indicate to the reader that the quotes are used as the evidence that are then interpreted and explicated, which aligns with inductive approaches to qualitative data analysis. We provide a more robust explanation of qualitative data collection and analysis in the methods section, heading ‘semi-structured interviews.’

d. General: I’m not confident that the data collected on a very discrete group is sufficient to report the findings outlined in this manuscript, especially noting the significant limitations that the authors themselves have identified.

Response: The gold standard in qualitative research is a sample of 12 participants. As per Guest et al. 2006, with a homogenous sample (e.g., veterans, deported, Latinos) qualitative data saturation can occur for the main research questions within 12-15 interviews. In the methods section, heading ‘semi-structured interviews’ we cite this work to illustrate how our work aligns with best practices in qualitative research.

While revising your submission, please upload your figure files to the Preflight Analysis and Conversion Engine (PACE) digital diagnostic tool, <https://pacev2.apexcovantage.com/>. PACE helps ensure that figures meet PLOS requirements. To use PACE, you must first register as a user. Registration is free. Then, login and navigate to the UPLOAD tab, where you will find detailed instructions on how to use the tool. If you encounter any issues or have any questions when using PACE, please email PLOS at [figures@plos.org](mailto:figures@plos.org). Please note that Supporting Information files do not need this step.

Please submit your revised manuscript by December 20th. If you will need more time than this to complete your revisions, please reply to this message or contact the journal office at [globalpubhealth@plos.org](mailto:globalpubhealth@plos.org). When you're ready to submit your revision, log on to <https://www.editorialmanager.com/pgph/> and select the 'Submissions Needing Revision' folder to locate your manuscript file.

Please include the following items when submitting your revised manuscript:

A rebuttal letter that responds to each point raised by the editor and reviewer(s). You should upload this letter as a separate file labeled 'Response to Reviewers'.

A marked-up copy of your manuscript that highlights changes made to the original version. You should upload this as a separate file labeled 'Revised Manuscript with Track Changes'.

An unmarked version of your revised paper without tracked changes. You should upload this as a separate file labeled 'Manuscript'.

Guidelines for resubmitting your figure files are available below the reviewer comments at the end of this letter.
