# Supplementary material for: Social determinants of health among noncitizen deported US veterans: A participatory action study
Source: PLOS Glob Public Health. 2023 Aug 2;3(8):e0002190. doi: 10.1371/journal.pgph.0002190 (PMC10396001; doi:10.1371/journal.pgph.0002190)
Supplement: Attachment — Submitted filename: Response to Reviewers.docx [file pgph.0002190.s010.docx]

**Response to Editors**

While the reviewers feel positive about the revisions on the manuscript, before we proceed further, please include a complete copy of PLOS’ questionnaire on inclusivity in global research in your revised manuscript. Our policy for research in this area aims to improve transparency in the reporting of research performed outside of researchers’ own country or community. The policy applies to researchers who have travelled to a different country to conduct research, research with Indigenous populations or their lands, and research on cultural artefacts. The questionnaire can also be requested at the journal’s discretion for any other submissions, even if these conditions are not met. Please find more information on the policy and a link to download a blank copy of the questionnaire here: https://journals.plos.org/plosone/s/best-practices-in-research-reporting. Please upload a completed version of your questionnaire as Supporting Information when you resubmit your manuscript.

- Response: We have completed this questionnaire.

Please review your reference list to ensure that it is complete and correct. If you have cited papers that have been retracted, please include the rationale for doing so in the manuscript text, or remove these references and replace them with relevant current references. Any changes to the reference list should be mentioned in the rebuttal letter that accompanies your revised manuscript. If you need to cite a retracted article, indicate the article’s retracted status in the References list and also include a citation and full reference for the retraction notice.

- Response: We reviewed the reference list and omitted the following references from the list as they were citations that aligned with text that was part of the previous manuscript but that text was removed during the revision process.

1. Fernández-Niño JA, Ramírez-Valdés CJ, Cerecero-Garcia D, Bojorquez-Chapela I. Deported Mexican migrants: health status and access to care. Rev Saude Publica. 2014;48(3):478-85. doi: 10.1590/s0034-8910.2014048005150. PubMed PMID: 25119943; PubMed Central PMCID: PMCPMC4203084.
2. Cheney AM, Newkirk C, Rodriguez K, Montez A. Inequality and health among foreign-born latinos in rural borderland communities. Soc Sci Med. 2018;215:115-22. Epub 20180911. doi: 10.1016/j.socscimed.2018.09.011. PubMed PMID: 30223174.
3. Pinedo M, Burgos JL, Ojeda VD. A critical review of social and structural conditions that influence HIV risk among Mexican deportees. Microbes Infect. 2014;16(5):379-90. Epub 20140226. doi: 10.1016/j.micinf.2014.02.006. PubMed PMID: 24583278; PubMed Central PMCID: PMCPMC4236109.
4. Bojorquez I, Aguilera RM, Ramírez J, Cerecero D, Mejía S. Common Mental Disorders at the Time of Deportation: A Survey at the Mexico-United States Border. J Immigr Minor Health. 2015;17(6):1732-8. doi: 10.1007/s10903-014-0083-y. PubMed PMID: 25118675.

We added the following citation as we realized this was missing:

1. Department of Veterans Affairs. Expanded Access to Non-VA Care Through the Veterans Choice Program. Final rule. *Fed Regist*. 2018;83(92):21893-21897.

**Reviewer's Responses to Reviewers**

**Reviewer #2**

Thank you for the extensive revisions. You have addressed my concerns with the manuscript.

- Response: We appreciate your positive feedback and thoughtful review.

One pedantic point (that does not effect my recommendation to publish): I would still like to see Table 1 a little clearer. eg three columns - Characteristics / n% / Mean (SD)

- Response: We have made Table 1 more clear by separate n(%) and mean (SD)

**Reviewer #3**

Thank you very much for the opportunity to review this revised manuscript on a participatory action study of noncitizen deported veterans’ experiences and health concerns. I found the revised submission to be highly responsive to reviewer comments on the previously submitted version of the manuscript.

- Response: Thank you for this positive feedback.

My only additional suggestions below are very minor recommended changes/corrections to several wording usages throughout the manuscript – please consider:

1. Throughout the manuscript:
Using only one of “sociodemographic” or “socio-demographic”

- Response: We used sociodemographic throughout.

2. Throughout the manuscript:
Using only one of “sociocultural” or “socio-cultural”

- Response: We used sociocultural throughout.

3. “Semi-structured Interviews” section, second paragraph, first sentence:
Clarifying how the first question probed about health

- Response: We revised this sentence to: “The interviewer first asked participants to share their thoughts on the following question: “What is it like to be a US veteran and a Mexican citizen having been deported from the US?” using probes to elicit additional information about participants’ experiences and understandings of the relationships between deportation and health.”

4. “Semi-structured Interviews” section, second-to-last sentence: Removing the second use of “interviews” from the sentence

- Response: Removed the second use of interviews.

5. Discussion section, first paragraph: Changing two instances of “see” to “saw”

- Response: We made these changes.

6. Discussion section, second paragraph, last sentence: Changing “veterans service history” to “veterans’ service history”

- Response: We made this change.

7. Conclusion section, second-to-last sentence: Inserting a word (“engaged”?) between “we” and “policy” in “we policy experts”

- Response: Added engaged to this sentence.

8. Conclusion section, last sentence: Inserting “be” before “studied”

- Response: Added “be” to this sentence.

9. Funding section, before last sentence: Removing quotation marks

- Response: Quote marks are removed.

While revising your submission, please upload your figure files to the Preflight Analysis and Conversion Engine (PACE) digital diagnostic tool, https://pacev2.apexcovantage.com/. PACE helps ensure that figures meet PLOS requirements. To use PACE, you must first register as a user. Registration is free. Then, login and navigate to the UPLOAD tab, where you will find detailed instructions on how to use the tool. If you encounter any issues or have any questions when using PACE, please email PLOS at figures@plos.org. Please note that Supporting Information files do not need this step.

- Response: We uploaded the files to this platform. See the snapshot below of the changes to the original files.
